# Long-Term Risk Assessment for Medical Application of Cold Atmospheric Pressure Plasma

**DOI:** 10.3390/diagnostics10040210

**Published:** 2020-04-11

**Authors:** Rico Rutkowski, Georg Daeschlein, Thomas von Woedtke, Ralf Smeets, Martin Gosau, Hans-Robert Metelmann

**Affiliations:** 1 Department of Oral and Maxillofacial Surgery, University Medicine Hamburg-Eppendorf, 20251 Hamburg, Germany; 2 Department of Dermatology, University Medicine Greifswald, 17475 Greifswald, Germany; 3 Leibniz Institute for Plasma Science and Technology (INP) e.V. Greifswald, 17489 Greifswald, Germany; 4 Department of Oral and Maxillofacial Surgery, Division of Regenerative Orofacial Medicine, University Medical Center Hamburg-Eppendorf, 20251 Hamburg, Germany; 5 Department of Oral and Maxillofacial Surgery/Plastic Surgery, University Medicine Greifswald, 17475 Greifswald, Germany

**Keywords:** wound healing, reactive oxygen species, cold atmospheric pressure plasma, hyperspectral imaging, confocal laser scanning microscopy

## Abstract

Despite increasing knowledge gained based on multidisciplinary research, plasma medicine still raises various questions regarding specific effects as well as potential risks. With regard to significant statements about in vivo applicability that cannot be prognosticated exclusively based on in vitro data, there is still a deficit of clinical data. This study included a clinical follow-up of five probands who had participated five years previously in a study on the influence of cold atmospheric pressure plasma (CAP) on the wound healing of CO_2_ laser-induced skin lesions. The follow-up included a complex imaging diagnostic involving dermatoscopy, confocal laser scanning microscopy (CLSM) and hyperspectral imaging (HSI). Hyperspectral analysis showed no relevant microcirculatory differences between plasma-treated and non-plasma-treated areas. In summary of all the findings, no malignant changes, inflammatory reactions or pathological changes in cell architecture could be detected in the plasma-treated areas. These unique in vivo long-term data contribute to a further increase in knowledge about important safety aspects in regenerative plasma medicine. However, to confirm these findings and secure indication-specific dose recommendations, further clinical studies are required.

## 1. Introduction

The use of cold atmospheric pressure plasma (CAP) for biomedical applications is the key element of plasma medicine [1]. This field of research, which is characterized by a high degree of interdisciplinarity and is located at the interface between natural and life sciences, has been attracting increasing scientific attention for several years [2]. With regard to its basic biological effectiveness, CAP is understood as a source of reactive oxygen (ROS) and nitrogen species (RNS), electromagnetic radiation and electric fields [3,4]. Depending on the plasma source, the elements of this "cocktail" are differently effective and can act as a combination of several therapeutic approaches. Both physiological and pathological cellular and subcellular processes communicate via ROS and RNS and can also be influenced or controlled by these effectors. Different cell types seem to have a different sensitivity to reactive species, which suggests the possibility of highly selective CAP effects. [3,5,6]. The target spectrum includes microbiological pathogens, intact and functionally deficient somatic as well as malignant cells [7]. A transfer of basic redox biological research, which still raises numerous questions, to biological effects indicates that low treatment intensities (short treatment times) have a cell-stimulating effect, while higher treatment intensities (longer treatment times) may cause inactivation and trigger apoptotic processes [8]. Numerous research groups are attempting to investigate and utilize these selective mechanisms of action for biomedical applications. Exemplary mention should be made here of the work of Xu et al. [9] on the influence of plasma-generated radicals on the structural modification of gap junctions and the research of Chen et al. [10] on CAP-mediated immune checkpoint blockade therapy in a transdermal approach. 

Redox active species also play an important role in wound-healing processes. In addition to a well-documented anti-infective efficacy, which includes bacterial wound pathogens as well as viruses, fungi, biofilms and multiresistant pathogens, and the fact they are becoming increasingly important in an ageing society heading towards a postantibiotic age, various CAP-mediated, dose-dependent effects on proliferative mechanisms, cell migration and angiogenesis have been demonstrated [11,12,13,14,15]. As a multidimensional therapeutic approach, innovative plasma technology could have an enormous potential in the field of wound healing and tissue regeneration and may reduce costs by eliminating protracted conventional treatments.

Intended for clinical use, aspects of risk and safety profiles are crucial. In this context, any short-, medium-, or long-term direct and indirect risks to patients, users and the environment must be analyzed critically and subjected to a detailed risk-benefit analysis. Despite all basic technological and practical progress, plasma medicine still raises some unresolved questions in this regard [16,17,18]. As a prediction of safety in vivo use based only on in vitro data is not sufficient for medical application, and a risk assessment outside basic experimental research in vitro or in animal studies is necessary, in particular long-term clinical studies. The aim of the present trial was to evaluate relevant clinical safety and risk aspects in the medical application of cold atmospheric pressure plasma based on a long-term study.

## 2. Materials and Methods

### 2.1. Study Population

Five human subjects (55, 34, 36, 45 and 67 years of age, which corresponds to a median age of 47.4 years) were examined within this study. All probands showed Fitz Patrick skin type II. The probands who had been part of a prospective, randomized, controlled study five years previously on the effect of cold atmospheric plasma on wound healing were subjected to a standardized follow-up. After inducing of four equal-sized (1 cm^2^) ablative laser lesions (CO_2_-Laser, UltraPulse^®^, Germany) as a single dose (20 W, 100 mJ, 200 pulses/min) in the area of the left forearm, lesions were treated with CAP within the original study. According to randomization, plasma treatment was performed once for 10 s, once for 30 s or on three consecutive days for 10 s each. The fourth lesion always remained untreated. CAP treatment was performed with kINPen^®^ MED plasma jet (INP Greifswald/neoplas tools GmbH, Greifswald, Germany). Setting, assessment and documentation of the wounds and the wound healing process was approved by the institutional ethics committee (Ethics vote: BB24/09 (2009), University Medicine Greifswald’s Ethics Committee). In follow-up, all probands showed a good general state of health without symptom-related restrictions of activity. There was no acute inflammation, irritation or other peculiarities of the skin in the test area. None of the probands underwent immunosuppressive or anti-infective local or systemic therapy in the study period. The work was carried out in accordance with standards of Helsinki Declaration following informed consent.

### 2.2. Follow-Up Setting

The follow-up examinations were carried out in the Department of Dermatology of University Medicine Greifswald by two specialists in dermatology with specific experience in tumor prevention and aftercare, without knowledge of the CAP scheme applied five years previously (blinded). No external agents, creams or other cosmetic products were applied one week before the examination. An acclimatization period of 15 min to spatial atmosphere characterized by a regulated humidity (50%) and temperature (21 °C) was given. 

### 2.3. Dermatoscopy

Dermatoscopy was performed using monoocular DermoGenius II^®^ (DermoScan GmbH, Regensburg, Germany). Investigations were evaluated according to ABCD rules of dermatoscopy by Stolz and Semmelmayer [19]. Total dermatoscopy score (TDS) was calculated from the partial scores taking into account criteria-specific factors. 

### 2.4. Hyperspectral Imaging

**Figure 1:**
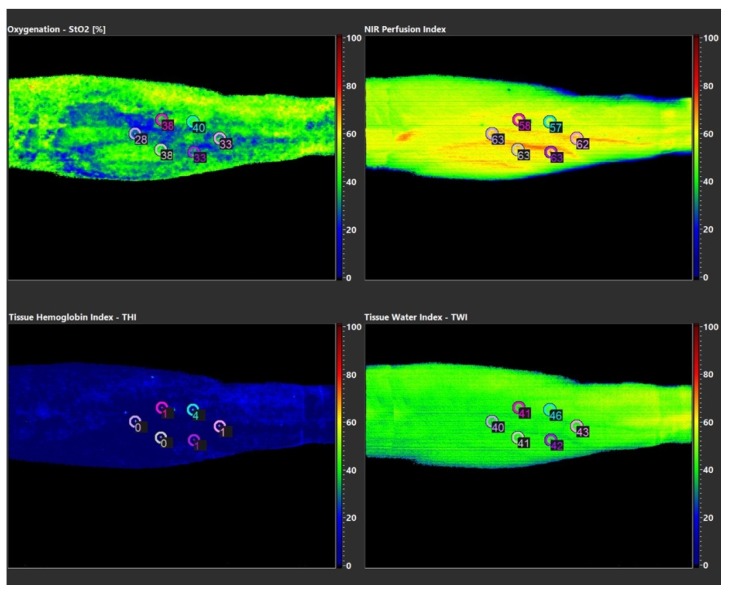
Visualized parameters of HSI measurements of proband 01.

Hyperspectral imaging (HSI) of plasma-treated skin areas was performed using TIVITA™ Tissue System (Diaspective Vision, Pepelow, Germany). Analyzable parameters include near-surface tissue oxygenation (StO2 in %), oxygenation in deep tissue layers (NIR perfusion, index value: 0–100), hemoglobin distribution (tissue hemoglobin index, THI, index value: 0–100) and tissue water index (TWI, index value: 0–100) that visualizes distribution of tissue water. After an acclimatization period, three measurements were taken from a distance of 0.5 m under completely darkened lighting conditions as recommended by the manufacturer and a 3 min pause between each measurement. Figure 1 shows the schematic arrangement of measuring points (point of interest, POI). Diagnostic potential of the TIVITA™ Tissue System for monitoring of microcirculatory CAP effects has been demonstrated in vivo [20,21].

### 2.5. Confocal Laser Scanning Microscopy

Examination was performed using a VivaScope 1500^®^ (Lucid Inc. Rochester, NY, USA and MAVIG GmbH, Munich, Germany), which uses an imaging wavelength of 830 nm. Lesions were systematically examined at a high-resolution level (horizontal image resolution <1.25 μm; vertical image resolution <5 μm) up to a depth of approximately 350 μm in real time, starting at epidermal surface. Physiological and pathological structural architectural, confocal microscopic criteria served as a reference. Physiological criteria were visualized on healthy skin before each examination (internal quality control).

### 2.6. Dermatology Life Quality Index (DLQI)

A possible impairment of quality of life was analyzed using Dermatology Life Quality Index (DLQI) [22]. Evaluation referred to the last seven days and, in sense of a separate (modified) evaluation, to a total period of five years, corresponding to the entire period since plasma treatment was completed. Due to the anatomically closely spaced lesions, a joint evaluation of overall area was carried out.

### 2.7. Patient and Observer Scar Assessment Scale (POSAS)

Skin structure in plasma-treated areas was evaluated using the Patient and Observer Scar Assessment Scale (POSAS) [23]. Assessment was performed individually for each of the four lesions of each proband.

## 3. Results

### 3.1. Dermatoscopy

None of the probands had a suspicious or malignant score in one of the four lesions. Subscores and total dermatoscopic score for all lesions are shown in Table 1.

### 3.2. Hyperspectral Imaging

StO2 measurements revealed intra- and interindividual differences up to 30%, depending on the point of interest (POI) examined. Index values measured for NIR and TWI showed intraindividual swings up to 13 index points and interindividual differences up to 19 index points (Table 2, Table 3, Table 4, Table 5 and Table 6). HSI parameters exemplary of a single measurement of proband 01 are shown in Figure 1. Microcirculatory parameters showed no causal relation to the plasma-treated areas, but seemed to follow anatomical conditions and structures (e.g., superficial skin veins). In summary, no relevant differences or pathologies between CA- treated and non-CAP-treated areas could be diagnosed with regard to analyzed microperfusion parameters.

### 3.3. Confocal Laser Scanning Microscopy 

**Figure 2:**
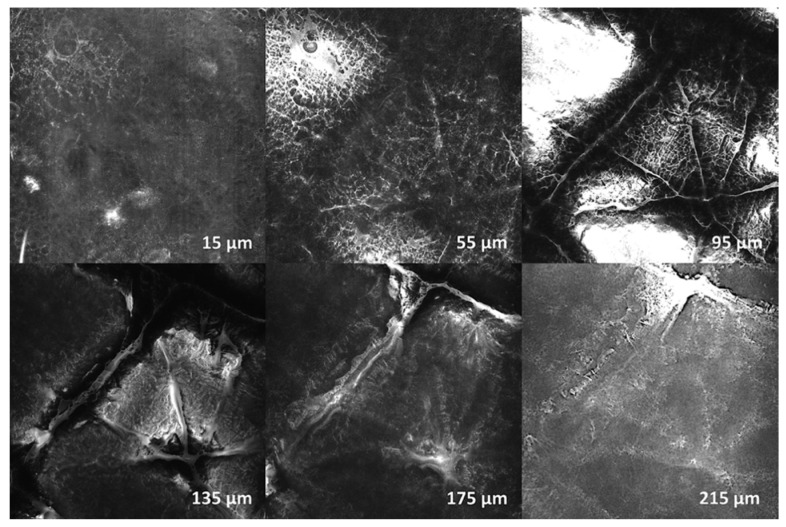
Confocal laser scanning microscopy (CLSM) examination of proband 01—exemplary shown for Lesion_02 (3 × 10 s CAP). Depth: 15, 55, 95, 135, 175, 215 µm. There were no signs of disturbed cell architecture or morphology in the sense of a cancerous or precancerous lesion, or any other pathological changes.

None of the skin areas treated with CAP showed signs of incipient or advanced tumor cell formation or other pathological changes between the epidermal surface and the corneal cell layer and stratum papillare of the dermis. The top layer (15 µm) showed corneal structures with reflective, relief-forming keratoses. Overall, a homogeneous cell architecture with regular cell boundaries and an inconspicuous internal structure without pathological changes was observed. In deeper skin layers (95–215 µm) numerous papillae with typically formed and distributed keratinocyte structures were found. In summary, it can be stated for all test persons that none of the investigated areas showed signs of a disturbed cell architecture or morphology, in sense of a cancerous or precancerous lesion or other pathological changes. No relevant differences between plasma-treated and untreated skin were found. Exemplary images of confocal laser scanning microscopy (CLSM) examinations of one lesion of proband 01 in different tissue depths are shown in Figure 2.

### 3.4. Dermatology Life Quality Index

Two separate periods were assessed, both the last 5 years since completion of CAP therapy (modified DLQI) and the previous 7 days (original DLQI). All probands rated each question with 0 points for both periods, which resulted in a total score of 0 points. Accordingly, no therapy-related impairment of quality of life could be determined.

### 3.5. Patient and Observer Scar Assessment Scale

For each lesion in all probands, a PSAS of six points (= minimum possible test result (6/60 points)) was determined. Additional evaluation of personal overall impression also resulted in the lowest possible value (1/10 points) (Table 7).

OSAS resulted in a total score of six points for probands 03 and 04. For probands 01, 02 and 05 there were differences both in the OSAS individual scores and in the separate overall evaluation. Lesion_01 (no CAP) of proband 01 showed minor hypopigmentation (3/10 points). For proband 02, hypopigmentation and surface contraction (2/10 points) in Lesion_04 (no CAP) and minor changes in pliability and relief (2/10 points) in Lesion_02 (1 × 10s CAP treatment) could be detected, compared to adjacent skin area. In proband 05 hypopigmentation and surface contraction (3/10 points) in lesion_02 (no CAP) and minor changes in pliability (2/10 points) and relief (3/10 points) in Lesion_04 (1 × 10 s CAP treatment) were observed (Table 8).

## 4. Discussion

In the present study, a systematic, clinical and state-of-the-art assisted long-term evaluation (5 years postinterventional) of a group of probands treated with cold atmospheric pressure plasma under standardized conditions was performed. The population analyzed corresponded to that of a study on the influence of CAP on wound healing of CO_2_ laser-induced skin lesions in the area of the left volar forearm that showed the best healing results after repetitive CAP application [24].

Dermatoscopy revealed no suspicious or malignant lesions. Hyperspectral analysis of superficial (StO2) and deep (NIR) tissue oxygen saturation, local hemoglobin distribution (THI) and tissue water content (TWI) showed no relevant microcirculatory differences between plasma-treated and non-plasma-treated areas. In confocal laser scanning microscopy, no precancerous or cancerous changes or signs of acute or chronic inflammatory reactions could be detected in any proband. In addition, no pathological modifications of cell architecture and cell morphology could be determined with CLSM compared to untreated skin. Using the Dermatology Life Quality Index, no therapy-associated impairments of quality of life were determined. Evaluation of plasma-mediated cutaneous side effects using the Patient and Observer Scar Assessment Scale (POSAS) showed a tendency towards increased values for lesions without treatment or with low-dose plasma treatment in some probands, especially from the examiner´s perspective (OSAS). 

Identification and evaluation of potential therapy-associated short- and long-term pathological effects is of great importance. From a physical perspective, electrical safety, application temperature and artificial optical radiation are of particular relevance for risk assessment. With regard to application temperature, a direct dependence on movement speed of the jet was proven, whereby about 40 °C was determined at a speed of 10 mm/s (≥ 8 mm/s = 35–45 °C) for kINPen^®^ MED [25]. By axial analysis, fiberoptic temperature measurements in a range between 35 and 40 °C were determined for the plasma effluent zone in direct contact with the tissue to be treated [26]. Furthermore, it was demonstrated that an ultraviolet dose with the endorsed treatment parameters is significantly below recommended exposure limits for incoherent radiation from artificial sources and even below minimal erythemal dose (MED), a level of ultraviolet (UV) dose that causes a reddening visible after 24 h [16,27,28].

In addition to physical aspects, complex interactions between plasma and tissue, in particular deoxyribonucleic acid (DNA), which are mainly mediated by reactive oxygen and nitrogen species (RONS), are a key research focus with regard to possible mutagenicity and genotoxicity. Numerous in vitro studies have proven dose-dependent plasma effects on isolated and cellular DNA. Different research groups focused on complex pathophysiological effects of CAP on isolated [29,30,31,32] and cellular [32,33,34,35] DNA. These findings, which were mainly collected in vitro, have already been partially supported by clinical data. While Isbary et al. [36] detected a dose-dependent increase of DNA damage in human skin ex vivo, Wu et al. [37] demonstrated a significant accumulation of γ-H2AX in healthy and damaged skin in an in vivo animal model, depending on CAP application duration. However, DNA damage must not necessarily have to be equated with a mutation. For example, detection of enriched γ-H2AX does not provide valid information about the type of DNA damage. In addition, an increase in γ-H2AX was also observed in the course of nongenotoxic apoptosis processes [38,39]. This requires an adequate differentiation between the actual mutagenic γ-H2AX potential and γ-H2AX formed during apoptosis. 

In addition to in vitro research, plasma-associated side effects and risk potential were also evaluated in animal experiments. Kos et al. [40] investigated direct and indirect damage mechanisms in a mouse model and concluded that plasma application (jet system) is not as safe as described by numerous other authors. However, critical analysis of this study shows various limitations. Tests combining low gas flow rates with short treatment periods were not performed. Taking into account the infrared images, which showed a surface temperature of 85 °C and higher, the detected damage can certainly be interpreted as classic burning effects and not isolated by an increased RONS formation. Due to more punctual skin damage, it can also be assumed that no movement of the jet has occurred. Treatment risk summarized by the authors should be transparently limited to corresponding parameters and preferably placed in the context of current treatment recommendations of approved devices. In contrast, various research groups were unable to demonstrate acute postinterventional side effects or malignant aspects in animal models [41,42,43,44,45]. Schmidt et al. [46] presented a first long-term analysis (1 year after completion of CAP therapy) on a mouse model and described both macroscopically and histologically inconspicuous results in plasma-treated wound areas without excessive scarring or chronic inflammatory reactions. Histologically and radiologically (magnetic resonance imaging, MRI) no malignant neoplasms could be detected neither in the area of the actual plasma treatment nor in selected organs.

However, there are several general and specific limitations. Similar to in vitro studies, various plasma concepts are also used in animal experiments, not all of which are approved for clinical use in accordance with European CE Regulation. Adequate comparability is also hampered by partly unpublished technical parameters and application schemes. A deficit compared to the common small animal models (especially mouse and rat) results from a thinner skin layer compared to humans and the fact that dermal wound healing, especially on the trunk, occurs primarily by contraction and not mainly by re-epithelisation processes, as it does in humans [42,47].

The risk assessment in most of the in vivo studies mainly focuses on acute side effects. However, longer observation periods are required for the clinical evaluation of long-term effects in terms of structural pathologies, mutagenicity and genotoxicity.

First case series and clinical studies on the application of cold plasma technology in humans already exist. Isbary et al. [48] investigated the use of cold atmospheric plasma for the treatment of chronically infected wounds in a prospective, randomized, controlled in vivo study. Besides a significant reduction in bacterial colonization in comparison to standard treatment, no side effects associated with CAP therapy were found after a total of 291 treatments. In the treatment of pruritus, skin graft sites and herpes zoster, no relevant side effects were registered either [49,50,51]. Brehmer et al. [52] reported on successful plasma therapy of chronic venous leg ulcers in a prospective, controlled clinical study using PlasmaDerm^®^. Metelmann et al. [24] published a clinical case series on the use of kINPen^®^ MED plasma jet in the early wound treatment of iatrogenic laser lesions in five volunteers. A total of 10 days after the initial laser treatment, the best result for repetitive, short-term plasma application (10 seconds on three consecutive days) was determined. Macroscopic follow-up examination published one year later showed no evidence of precancerous or cancerous lesions in the plasma-treated skin areas [53].

Most in vivo research focuses on antimicrobial efficacy and therapy of infectious skin diseases. Questions regarding long-term risk and safety aspects in particular still play a subordinate role. Most of these studies also lack an objective, side-effect-oriented base for evaluation. The results of this study support the lack of local side effects described in the in vivo studies conducted so far. However, there are various limitations to be considered. Due to different technical concepts, this statement cannot be generalized to all existing plasma devices and process parameters. Even for the device originally used (kINPen^®^ MED), the statement must be limited to the application schemes used, taking into account the still scarce long-term clinical data. The standardized, iatrogenic laser wounds allow both inter- and intraindividual comparability, but include laser treatment itself as a factor to be considered. A general limitation also results from the low number of probands, which does not permit an adequately significance analysis nor differentiated considerations with regard to gender- or age-specific as well as comorbidity-associated differences. 

## 5. Conclusions

An essential prerequisite for complete academic, clinical but also socioeconomic acceptance of a therapeutic procedure is, in addition to an evidence-based efficacy itself, the scientifically based analysis of potential risks and side effects. The general question of plasma-associated risk potential feeds central and highly up-to-date discussions. In our aging society, which is heading towards a postantibiotic era, with increasing challenges such as multimorbidity, polypharmacy and globally progressive resistance problems of conventional antibiotics, anti-infective use is currently the best researched field of CAP application. In the present study, it could be shown that the local application of cold atmospheric plasma (considering the application schemes used in the initial study), especially with regard to therapy-associated formations of cancerous or precancerous lesions and local pathological influence on cutaneous microcirculation, is to be classified as safe. From the point of view of biological safety, future clinical studies could include the evaluation of possible long-term effects in the context of practice-based application schemes over a longer period of time. Furthermore, a differentiated consideration of locoregional and systemic conditions is required (e.g., intact skin vs. wound vs. tumor tissue; skin vs. musculature vs. nerve tissue; immunocompetent vs. immunocompetent organism). Especially for clinical physicians, who are not educated in plasma physics, the sum of basic in vitro and in vivo research should ultimately lead to indication-specific doses and treatment windows.

## Figures and Tables

**Table 1:** Dermatoscopic evaluation of probands 01-05, TDS is shown for each lesion.

TDS	no CAP	1 × 10 s CAP	3 × 10 s CAP	1 × 30 s CAP
Proband 01	2.7	1.5	1	1
Proband 02	2.1	1	1	1.5
Proband 03	1.6	1	1	1
Proband 04	1	1	1	1
Proband 05	3.5	1.5	1	1

**Table 2:** Hyperspectral imaging (HSI). Mean values ± variance and standard deviation (s), based on three individual HSI measurements on proband 01.

Proband 01	Oxygenation (StO2, %)	Perfusion Index (NIR)	Tissue Hemoglobin Index (THI)	Tissue Water Index (TWI)
Lesion_01 (no CAP)	40.67 ± 0.89 s = 0.94	56.00 ± 0.67 s = 0.82	3.67 ± 0.22 s = 0.47	44.33 ± 2.89 s = 1.70
Lesion_02 (3 × 10 s)	33.67 ± 0.22 s = 0.47	63.00 ± 0.67 s = 0.82	1.33 ± 0.22 s = 0.47	41.00 ± 0.67 s = 0.82
Lesion_03 (1 × 30 s)	40.33 ± 4.22 s = 2.05	59.00 ± 0.67 s = 0.82	0.67 ± 0.22 s = 0.47	42.00 ± 2.00 s = 1.41
Lesion_04 (1 × 10 s)	38.33 ± 0.22 s = 0.47	62.67 ± 1.56 s = 1.25	1.00 ± 0.67 s = 0.82	41.67 ± 0.89 s = 0.94
Proximal Control	33.67 ± 0.22 s = 0.47	62.67 ± 0.89 s = 0.94	0.67 ± 0.22 s = 0.47	41.33 ± 1.56 s = 1.25
Distal Control	29.00 ± 0.67 s = 0.82	63.33 ± 0.22 s = 0.47	0.33 ± 0.22 s = 0.47	40.00 ± 0.00 s = 0.00

**Table 3:** Hyperspectral imaging. Mean values ± variance and standard deviation (s), based on three individual HSI measurements on proband 02.

Proband 02	Oxygenation (StO2, %)	Perfusion Index (NIR)	Tissue Hemoglobin Index (THI)	Tissue Water Index (TWI)
Lesion_01 (1 × 30 s)	31.67 ± 1.70 s = 1.70	50.67 ± 0.22 s = 0.47	0.33 ± 0.22 s = 0.47	43.33 ± 0.89 s = 0.94
Lesion_02 (1 × 10 s)	37.33 ± 4.22 s = 2.05	54.67 ± 1.56 s = 1.25	1.33 ± 0.22 s = 0.47	41.33 ± 2.89 s = 1.70
Lesion_03 (3 × 10 s)	35.00 ± 0.67 s = 0.82	53.33 ± 0.89 s = 0.94	0.33 ± 0.22 s = 0.47	44.00 ± 0.67 s = 0.82
Lesion_04 (no CAP)	26.67 ± 2.89 s = 1.70	56.67 ± 0.22 s = 0.47	0.33 ± 0.22 s = 0.47	38.00 ± 0.67 s = 0.82
Proximal Control	24.33 ± 1.56 s = 1.25	57.33 ± 0.22 s = 0.47	0.67 ± 0.22 s = 0.47	44.33 ± 2.89 s = 1.70
Distal Control	22.33 ± 1.56 s = 1.25	57.00 ± 0.00 s = 0.00	0.67 ± 0.89 s = 0.94	34.67 ± 1.56 s = 1.25

**Table 4:** Hyperspectral imaging. Mean values ± variance and standard deviation (s), based on three individual HSI measurements on proband 03.

Proband 03	Oxygenation (StO2, %)	Perfusion Index (NIR)	Tissue Hemoglobin Index (THI)	Tissue Water Index (TWI)
Lesion_01 (1 × 30 s)	16.67 ± 0.22 s = 0.47	47.67 ± 2.89 s = 1.70	0.33 ± 0.22 s = 0.47	45.33 ± 1.56 s = 1.25
Lesion_02 (1 × 10 s)	33.33 ± 0.89 s = 0.94	51.33 ± 2.89 s = 1.70	0.00 ± 0.00 s = 0.00	47.67 ± 4.22 s = 2.05
Lesion_03 (no CAP)	17.00 ± 2.00 s = 1.41	47.67 ± 0.22 s = 0.47	0.00 ± 0.00 s = 0.00	41.33 ± 0.89 s = 0.94
Lesion_04 (3 × 10 s)	24.33 ± 4.22 s = 2.05	50.00 ± 0.00 s = 0.00	0.67 ± 0.22 s = 0.47	42.33 ± 1.56 s = 1.25
Proximal Control	44.33 ± 1.56 s = 1.25	50.67 ± 0.22 s = 0.47	0.33 ± 0.22 s = 0.47	44.00 ± 0.67 s = 0.82
Distal Control	14.33 ± 2.89 s = 1.70	55.67 ± 1.56 s = 1.25	0.33 ± 0.22 s = 0.47	34.67 ± 0.89 s = 0.94

**Table 5:** Hyperspectral imaging. Mean values ± variance and standard deviation (s), based on three individual HSI measurements on proband 04.

Proband 04	Oxygenation (StO2, %)	Perfusion Index (NIR)	Tissue Hemoglobin Index (THI)	Tissue Water Index (TWI)
Lesion_01 (1 × 30 s)	36.67 ± 0.89 s = 0.94	50.67 ± 0.22 s = 0.47	0.67 ± 0.22 s = 0.47	34.00 ± 0.00 s = 0.00
Lesion_02 (1 × 10 s)	21.33 ± 4.22 s = 2.05	51.33 ± 1.56 s = 1.25	0.33 ± 0.22 s = 0.47	32.33 ± 0.89 s = 0.94
Lesion_03 (3 × 10 s)	40.33 ± 4.22 s = 2.05	48.33 ± 0.22 s = 0.47	5.33 ± 0.22 s = 0.47	34.33 ± 0.89 s = 0.94
Lesion_04 (no CAP)	33.00 ± 2.00 s = 1.41	49.33 ± 0.22 s = 0.47	1.00 ± 0.00 s = 0.00	36.67 ± 0.89 s = 0.94
Proximal Control	36.00 ± 0.67 s = 0.82	49.66 ± 2.89 s = 1.70	0.67 ± 0.22 s = 0.47	34.67 ± 0.22 s = 0.47
Distal Control	28.33 ± 1.56 s = 1.25	51.67 ± 0.22 s = 0.47	1.00 ± 0.00 s = 0.00	34.67 ± 2.89 s = 1.70

**Table 6:** Hyperspectral imaging. Mean values ± variance and standard deviation (s), based on three individual HSI measurements on proband 05.

Proband 05	Oxygenation (StO2, %)	Perfusion Index (NIR)	Tissue Hemoglobin Index (THI)	Tissue Water Index (TWI)
Lesion_01 (3 × 10 s)	43.33 ± 0.89 s = 0.94	50.67 ± 0.22 s = 0.47	6.67 ± 0.22 s = 0.47	41.00 ± 2.00 s = 1.41
Lesion_02 (no CAP)	42.67 ± 2.89 s = 1.70	53.67 ± 0.22 s = 0.47	2.67 ± 1.56 s = 1.25	38.33 ± 2.89 s = 1.70
Lesion_03 (1 × 30 s)	31.67 ± 0.89 s = 0.94	59.67 ± 1.56 s = 1.25	0.67 ± 0.22 s = 0.47	37.33 ± 0.89 s = 0.94
Lesion_04 (1 × 10 s)	39.00 ± 2.67 s = 1.63	55.00 ± 2.00 s = 1.41	1.00 ± 0.00 s = 0.00	42.67 ± 1.56 s = 1.25
Proximal Control	54.33 ± 0.22 s = 0.47	52.33 ± 0.89 s = 0.94	2.33 ± 0.22 s = 0.47	49.33 ± 1.56 s = 1.25
Distal Control	33.67 ± 1.56 s = 1.25	53.00 ± 0.00 s = 0.00	1.67 ± 0.22 s = 0.47	37.33 ± 0.22 s = 0.47

**Table 7:** Patient Scar Assessment Scale (PSAS) results for probands 01-05. Individual scores, total score and separate overall assessment are shown.

Parameter	Proband 01	Proband 02	Proband 03	Proband 04	Proband 05
Pain	1	1	1	1	1
Itching	1	1	1	1	1
Color	1	1	1	1	1
Stiffness	1	1	1	1	1
Thickness	1	1	1	1	1
Irregularity	1	1	1	1	1
Total Score	6	6	6	6	6
Overall Assessment	1	1	1	1	1

**Table 8:** Observer Scar Assessment Scale (OSAS) results for probands 01-05. Individual scores, total score and separate overall assessment are shown.

Parameter	Proband 01	Proband 02	Proband 03	Proband 04	Proband 05
Vascularity	1	1	1	1	1
Pigmentation	3 *	2 *	1	1	3 *
Thickness	1	1	1	1	1
Relief	1	2 *	1	1	3 *
Pliability	1	2 *	1	1	2 *
Surface Area	1	2 *	1	1	3 *
Total Score	8 *	10 *	6	6	13 *
Overall Assessment	2 **	2 **	1	1	2 **

* The highest individual score specific to the lesion and the total score resulting from the corresponding sum are shown. ** Shown is the highest overall rating for a specific lesion.

## Abbreviations

CAP	Cold atmospheric (pressure) plasma
HSI	Hyperspectral imaging
CLMS	Confocal laser scanning microscopy
POSAS	Patient and Observer Scar Assessment Scale
ROS	Reactive oxygen species
RNS	Reactive nitrogen species
TDS	Total dermatoscopic score
POI	Point of interest
StO2	Oxygenation (%)
NIR	Perfusion Index
THI	Tissue Hemoglobin Index
TWI	Tissue Water Index
DLQI	Dermatology Life Quality Index
MED	Minimal erythemal dose
DNA	Deoxyribonucleic acid
UV	Ultraviolet
MRI	Magnetic Resonance Imaging
